# BonMOLière: Small-Sized Libraries of Readily Purchasable Compounds, Optimized to Produce Genuine Hits in Biological Screens across the Protein Space

**DOI:** 10.3390/ijms22157773

**Published:** 2021-07-21

**Authors:** Neann Mathai, Conrad Stork, Johannes Kirchmair

**Affiliations:** 1 Computational Biology Unit (CBU) and Department of Chemistry, University of Bergen, N-5020 Bergen, Norway; neann.mathai@uib.no; 2 Center for Bioinformatics (ZBH), Department of Informatics, Universität Hamburg, 20146 Hamburg, Germany; stork@zbh.uni-hamburg.de; 3 Division of Pharmaceutical Chemistry, Department of Pharmaceutical Sciences, University of Vienna, 1090 Vienna, Austria

**Keywords:** optimized compound library, biological screening, purchasable compounds, evolutionary optimization, genetic algorithms, tool compounds, novel targets

## Abstract

Experimental screening of large sets of compounds against macromolecular targets is a key strategy to identify novel bioactivities. However, large-scale screening requires substantial experimental resources and is time-consuming and challenging. Therefore, small to medium-sized compound libraries with a high chance of producing genuine hits on an arbitrary protein of interest would be of great value to fields related to early drug discovery, in particular biochemical and cell research. Here, we present a computational approach that incorporates drug-likeness, predicted bioactivities, biological space coverage, and target novelty, to generate optimized compound libraries with maximized chances of producing genuine hits for a wide range of proteins. The computational approach evaluates drug-likeness with a set of established rules, predicts bioactivities with a validated, similarity-based approach, and optimizes the composition of small sets of compounds towards maximum target coverage and novelty. We found that, in comparison to the random selection of compounds for a library, our approach generates substantially improved compound sets. Quantified as the “fitness” of compound libraries, the calculated improvements ranged from +60% (for a library of 15,000 compounds) to +184% (for a library of 1000 compounds). The best of the optimized compound libraries prepared in this work are available for download as a dataset bundle (“BonMOLière”).

## 1. Introduction

A key strategy to identify bioactive compounds for biomacromolecules of interest is to screen large collections of compounds with biochemical or cell-based assays [1]. The success of such screening campaigns depends on many factors, above all, the quality and composition of the compound library: the much-cited “needle in the haystack” can only possibly be found if it actually is in the haystack. For this reason, the design of compound libraries for screening has been, and continues to be, an active field of research [2,3,4].

There are multiple approaches to compiling compound libraries. Focused design aims to compile a set of compounds that have an increased likelihood of being active on a particular target of interest [4,5,6,7]. In contrast, general compound libraries may be optimized for maximum chemical and/or biological diversity in order to increase the chances of identification of bioactive compounds for an arbitrary target [4,5,6,7,8]. In any case, besides chemical and biological diversity, the potential of compounds to be further optimized into functional molecules with desired properties (drugs, agrochemicals, or active cosmetic ingredients, in particular) should be taken into account [9]. In particular, this concerns the chemistry and toxicological properties of compounds. A high-quality compound library should also not include compounds that are prone to cause false readouts in biological assays (“bad actors”) [10,11].

With the ever-growing capacities in experimental screening, and in particular with the renewed interest in phenotypic screens, there have been increased efforts in compiling diverse compound libraries [8,9,12,13,14]. Lahue et al. [9] detail the recent undertaking at Merck & Co. to design two libraries for phenotypic screening, consisting of 50 thousand and 250 thousand compounds, respectively. They used a combination of in-house structural alerts and PAINS patterns [15] to remove compounds that contain undesired moieties. Chemical properties of compounds, such as the molecular weight and the quantitative estimate of drug-likeness score [16] (QED; a composite score to quantify chemical beauty), were used to filter and reduce the size of the candidate pool. The candidate pool was then organized into clusters, from which compounds were randomly selected to make compound sets using a genetic algorithm. The compound sets were improved by maximizing a fitness function that captured the 3D shape diversity, bioactivity diversity, and the median QED score of a set of compounds. The optimized sets were added to the final compound library after additional quality checks and opinions garnered from in-house chemists.

Schuffenhauer et al. [17] described the process used to design the screening deck at Novartis to optimize the selection of diverse subsets for screening. Structures were first passed through quality checks, frequent hitter SMARTS patterns (based on a subset of the PAINS patterns and in-house patterns to flag nuisance compounds), molecular weight, and nitrogen and oxygen contents. The compounds were flagged based on these checks and then ranked based on aqueous solubility, cell permeability, and the number of assigned flags from the checks. Additionally, compounds were grouped, non-exclusively, into classes that described their target interaction, biological and chemical descriptors. Compounds were selected in iterative rounds in a greedy fashion, starting with the highest ranking compound which occupied the highest number of classes. This selection, based on property ranking and the class memberships of the compounds, resulted in a 2D grid with 1.5 million compounds. The *x*-axis of the grid represents the property rank and the *y*-axis represents the round in which the compound was selected. The 2D grid allows the researchers at Novartis to select their choice of how many compounds to screen from this grid, balancing the properties and diversity of the screening subset.

A small to medium-sized screening deck (1000 to 15,000 compounds) that has a high chance of producing genuine hits during screening on an arbitrary target of interest can be incredibly valuable to biochemical and cell research. This is particularly true for the research of proteins for which there is little existing knowledge to use for the compilation of focused screening sets, and for academic research which is often focused on innovative targets under tight resource constraints.

**Figure 1:**
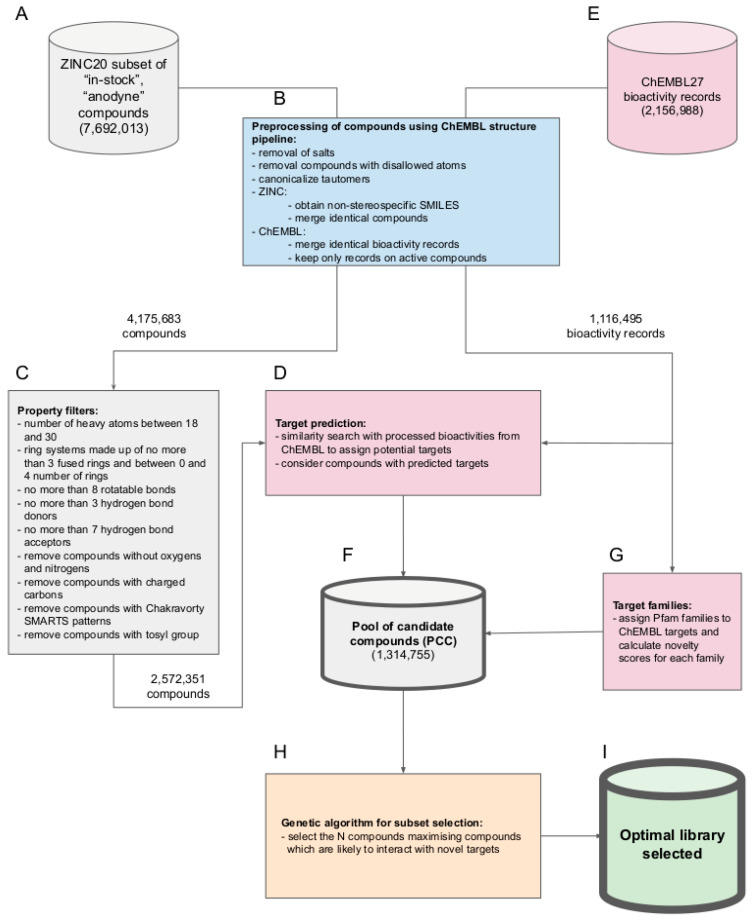
Overview of the workflow followed to generate optimized compound libraries: (**A**) source of compounds for the generation of optimized screening libraries, (**B**) preprocessing of compounds, (**C**) removal of compounds with undesired properties, (**D**) target prediction, (**E**) source of bioactivity data for target prediction, (**F**) ZINC20 compounds with predicted targets, (**G**) assignment of Pfam families, (**H**) genetic algorithm for optimal subset selection, (**I**) optimal library selected.

In this work, we report on the development and application of a computational method for the automated compilation of small to medium-sized compound libraries that have a high chance of producing genuine hits during experimental screens on arbitrary protein targets. We show the capacity of the new computational approach by generating a set of optimized compound libraries (“BonMOLière”) of different sizes from a subset of the ZINC20 database [18,19] (an aggregate of more than 300 commercially available compound catalogs from over 150 companies; Figure 1A). The approach utilizes (i) elaborate protocols for the preprocessing and preparation of chemical and biological data (Figure 1B), (ii) established rule sets to promote drug-likeness (Figure 1C), (iii) a validated, similarity-based approach to predict the likely targets of compounds (Figure 1D,E), and (iv) a genetic optimization algorithm (Figure 1H) to maximize coverage of the protein space (Figure 1G) when selecting subsets of compounds for a compound library, taking target novelty and target diversity into account.

## 2. Results and Discussion

All 7,692,013 compounds included in the ZINC20 subset used in this study (Figure 1A) fulfill the following key criteria (among other criteria, outlined in the Materials and Methods section):

1. The compounds are already made and readily obtainable from the manufacturer (i.e., they are part of the “in-stock” subset of the ZINC20 database).
2. The compounds are presumed benign in the context of screening with biological assays (i.e., they are also part of the “anodyne” subset of the ZINC20 database). More specifically, all of these compounds have passed an extensive collection of reactivity filters and PAINS patterns compiled and utilized by the developers of the ZINC database, meaning that they are unlikely reactive or causing pan-assay interference [20].

A multi-step process including the preparation of chemical and biological data (Figure 1B), the filtering for physicochemical properties to promote drug-likeness (Figure 1C), and the prediction of likely targets with a 2D similarity-based approach (Figure 1D), resulted in a pool of (1,314,755) candidate compounds (PCC). On this PCC, a genetic algorithm is applied to generate the final set of optimized screening libraries.

### 2.1. Characterization of the Pool of Candidate Compounds

In order to understand the relevance and properties of the PCC, and to enable a comparison of compound libraries prior and after optimization, we conducted a thorough characterization of the PCC.

#### 2.1.1. Physicochemical and Structural Characterization of the Pool of Candidate Compounds

**Figure 2:**
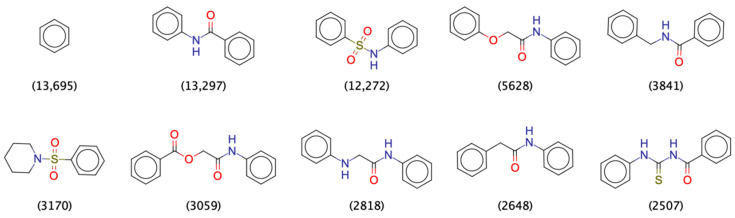
Top ten most popular Murcko scaffolds among the pool of candidate compounds. The numbers in the parentheses indicate how many compounds (out of 1,314,755) in the PCC have the scaffold.

The PCC consists of 1,314,755 compounds which are built on 379,690 unique Murcko scaffolds. Ninety-six percent of the scaffolds (362,707 scaffolds) represent fewer than ten compounds of the PCC. However, the remaining 16,983 scaffolds (4% of all scaffolds) have a wide distribution in terms of occurrence, ranging from 10 to 13,695 compounds per scaffold. The 10 most popular scaffolds (Figure 2) account for 60,428 compounds, with benzene being the most popular one (representing 13,695 or 1% of the PCC). Clustering of the PCC (with the Taylor-Butina algorithm and a Tanimoto similarity threshold of 0.4; see Materials and Methods) produced 28,826 clusters, 6801 of which are singletons.

**Figure 3:**
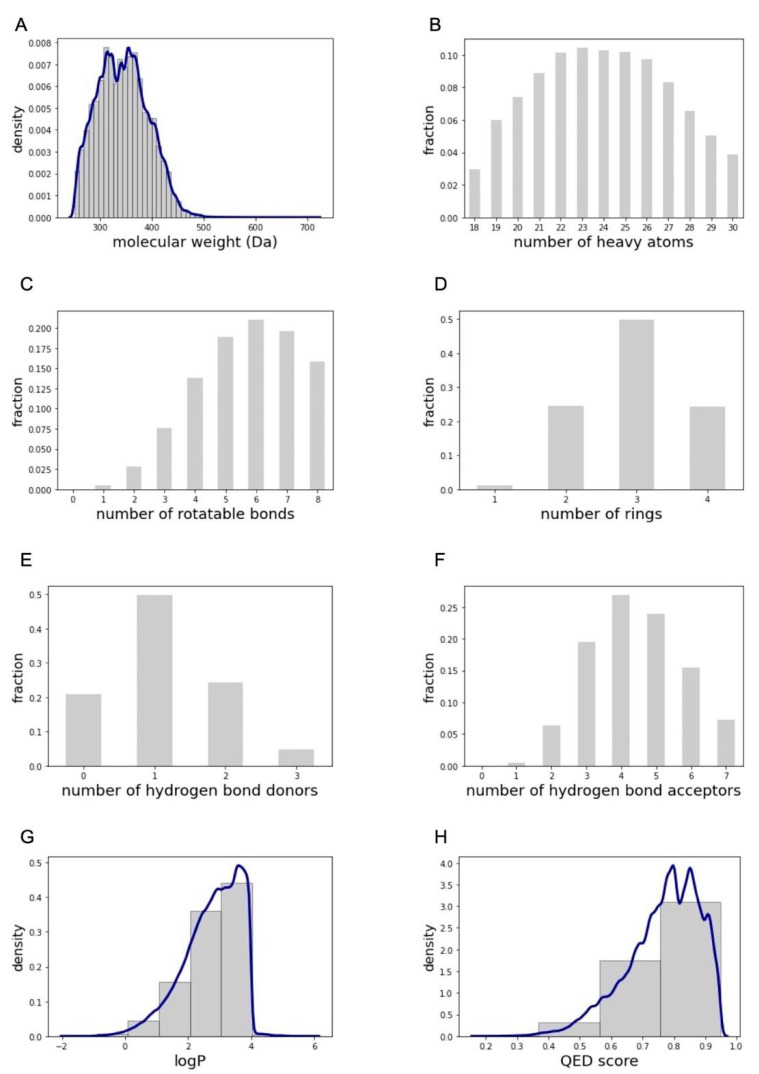
Distributions of physicochemical properties observed for the PCC: (**A**) molecular weight, (**B**) number of heavy atoms, (**C**) number of rotatable bonds, (**D**) number of rings, (**E**) number of hydrogen bond donors (**F**) number of hydrogen bond acceptors, (**G**) logP (note that for a very few compounds the logP value is greater than 4; this is because these logP values are calculated with RDKit (version 2020.09.1.0) [26] and may differ, to some extent, from the calculated logP values provided in the ZINC20 database), and (**H**) QED score.

The upper and lower boundaries of several relevant physicochemical properties of the compounds forming the PCC are set by the property filters applied previously (Figure 1C). In the case of the molecular weight, the property filters imposed an upper limit of 900 Da. The majority of the compounds forming the PCC have a molecular weight between 250 and 500 Da (Figure 3A), with the median at 342 Da. The median number of heavy atoms is 24 (Figure 3B). The majority of the compounds have 6 to 8 rotatable bonds (Figure 3C) and their median number of rings is 3 (Figure 3D). Half of all compounds have 1 hydrogen bond donor (Figure 3E) while the number of hydrogen bond acceptors per compound is more spread out (median at 4 hydrogen bond acceptors; Figure 3F). The distribution of the logP values of the compounds shows a peak near the upper filter boundary (Figure 3G), at approximately 4, with the median located at 2.90. Finally, while not utilized as a physicochemical property filter, Figure 3H shows the distribution of the QED score [16]. The QED score is a quantification of the drug-likeness of a compound, with 0 being most unfavorable and 1 being most favorable. The compounds of the PCC have a QED score distribution which is skewed towards being favorable, with a median score of 0.78. This shows that the PCC is composed of compounds with a high level of drug-likeness.

#### 2.1.2. Biological Characterization of the Pool of Candidate Compounds

**Figure 4:**
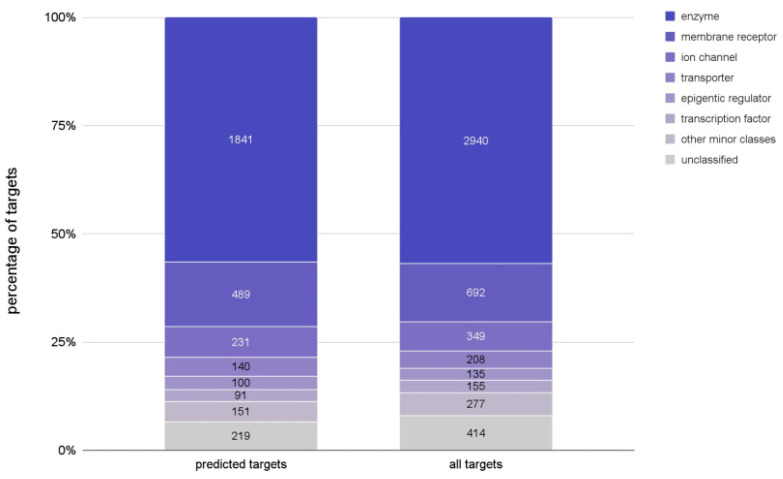
Types of proteins among the 3362 targets predicted for the ZINC20 compounds and the 5170 targets in the ChEMBL27 reference set. The size of the bars reflects the percentage of a target type represented while the labels are the counts of the targets for each type.

For the compounds forming the PCC, activities on a total of 3262 distinct protein targets were predicted with a target prediction model based on 2D molecular similarity. This model has been published and validated previously [21] and is built on a curated subset of the ChEMBL27 database [22,23] (the “ChEMBL27 reference set”) that covers a total of 5170 proteins (see Materials and Methods). As shown in Figure 4, the types of targets (enzymes, membrane receptors, etc.) predicted for the PCC are a good reflection of the proteins represented in the ChEMBL27 reference set.

To characterize the target diversity of compound libraries, we retrieved the Pfam family classifications [24,25] of the ChEMBL proteins by scanning their sequences against the Pfam database of 18,259 families. The compounds of the PCC were predicted to be active on 3262 unique targets that represent 880 Pfam families. These predicted targets are diverse and cover over 70% of the 1214 Pfam families that represent the 5170 proteins in the ChEMBL27 reference set. Of the proteins represented in the ChEMBL27 reference set, 334 belong to more than one Pfam family and two proteins are assigned to the dummy Pfam family to group all targets for which a Pfam family could not be assigned.

A novelty score for each Pfam family was calculated based on how many ChEMBL bioactivities were recorded before and after the year 2010 (Equation (1)). The intention of this score is to promote, during compound library optimization, the representation of protein targets that reflect the more recent research directions (as the protein space and the chemical space of interest evolve over time). The novelty scores were assigned to the proteins via their Pfam classification:

(1) $$\begin{array}{c} \mathrm{Pfam}\;\mathrm{novelty}\;\mathrm{score}\;=\\ \frac{\mathrm{No}.\left(\mathrm{bioactivities}\;\mathrm{recorded}\;\mathrm{in}\;\mathrm{or}\;\mathrm{after}\;2010\right)}{\mathrm{No}.\left(\mathrm{bioactivities}\;\mathrm{recorded}\;\mathrm{in}\;\mathrm{or}\;\mathrm{after}\;2010\right)+\mathrm{No}.\left(\mathrm{bioactivities}\;\mathrm{recorded}\;\mathrm{before}\;2010\right)} \end{array}$$

**Figure 5:**
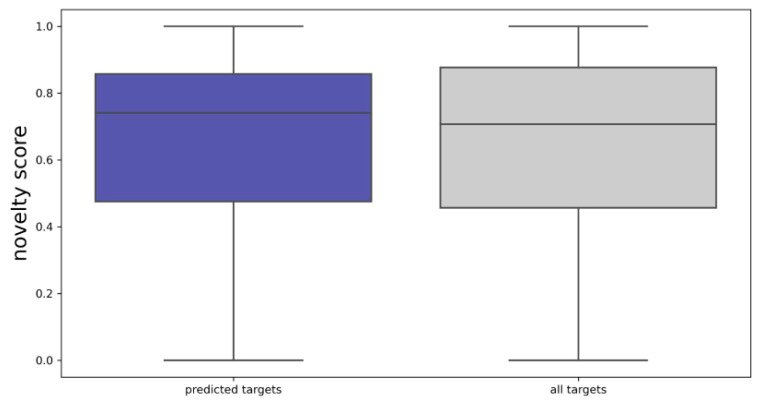
Distribution of novelty scores of the targets predicted for the PCC and of all targets found in the ChEMBL27 reference set.

The distribution of the novelty scores (Figure 5) of the predicted targets of the PCC closely mirrors the distribution of the novelty scores of the full protein space of the ChEMBL27 reference set, with median novelty scores of 0.74 and 0.71, respectively. This indicates that the predicted targets are generally representative of the proteins found in the ChEMBL27 reference set, with a slight bias towards newer targets.

### 2.2. Characterization of the Optimized Compound Libraries

Starting from the PCC, optimized compound libraries composed of 1000, 5000, 10,000, and 15,000 compounds were generated with the genetic algorithm described in the Materials and Methods section. As this is a subset selection problem, it is an optimization problem as an optimal subset needs to be selected which may be achieved through numerous combinations of compounds. A genetic algorithm is well suited for this as it reaches an optimal selection by selecting compounds to maximize the values of the fitness function. The algorithm optimizes the fitness of a compound set (i.e., a subset of the PCC) according to a fitness function that accounts not only for the novelty of the proteins predicted as targets of the compounds in a set but also for the number of proteins and number of times (i.e., the count) specific Pfam families are predicted for the compounds of a library (Equation (2)). This is because the more times a Pfam family is assigned to a library, via predicted targets for the compounds within a library, the higher the likelihood that proteins within this family will be a true hit when screened with this library.

(2) $$\mathrm{fitness}\;\mathrm{score}\;=\frac{\left(\sum_{}^{\;\mathrm{Pfam}\;\mathrm{family}}\mathrm{Pfam}\;\mathrm{novelty}\;\mathrm{score}\left(\frac{1-0.99^{\mathrm{count}}}{1-0.99}\right)\right)}{\mathrm{number}\;\mathrm{of}\;\mathrm{compounds}\;\mathrm{in}\;\mathrm{the}\;\mathrm{library}}$$

Therefore, when comparing two libraries of the same size, a higher fitness score signifies a better library, enriched with:

1. more bioactive compounds as a whole
2. compounds active on proteins representing more Pfam families (maximizing target diversity)
3. compounds active on newer targets (the novelty score is higher for newer targets)

#### 2.2.1. Baseline Compound Libraries

To understand the benefits of this approach (i.e., utilizing a target prediction model and a genetic algorithm to optimize the selection of compounds for the libraries), baseline compound libraries (of sizes 1000, 5000, 10,000, and 15,000) were generated. These baseline compound libraries were generated by randomly selecting sets of compounds from the 2,572,351 ZINC20 compounds which passed through the property filters (Figure 1C) irrespective of whether they are a part of the PCC or not. The selection was repeated multiple (10% of the compound library size) times and the properties of the fittest of these compound libraries are reported in Table 1.

#### 2.2.2. Optimized Compound Libraries

The genetic algorithm was run for 300 generations, with a population consisting of individual compound sets. The number of individuals for a population was set to 10% of the size of the library being optimized. The population evolved to reflect the fittest individuals from the previous generation (see Materials and Methods).

**Figure 6:**
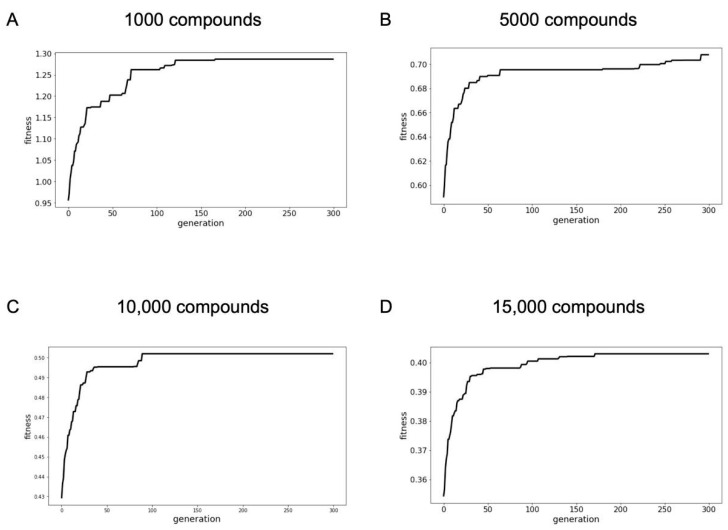
Development of the fittest population over 300 generations of a library of (**A**) 1000 compounds, (**B**) 5000 compounds, (**C**) 10,000 compounds, and (**D**) 15,000 compounds.

Within the 300 generations of evolution, the fitness increased (Table 2) and converged (Figure 6) for compound libraries of all sizes, with the biggest effects observed for the smallest compound library. That is, the fitness of the 1000-compound library improved by 34%, whereas the fitness of the 15,000-compound library increased by only 14% through the course of evolution. This shows that there are greater gains in optimization using this genetic algorithm for the smaller libraries than for larger libraries. It must be noted that, unlike the percentage improvement, the fitness score (Equation (2)) is a function of the number of compounds in a library and can therefore only be compared when they describe libraries of the same size and not libraries with different numbers of compounds.

By the similarity principle, a more diverse compound library should reflect a more diverse set of targets on which compounds of that library are active. The similarity principle holds true for the 5000-compound library: looking at the 5000-compound library (Table 2), we see that over the 300 generations the number of Murcko scaffolds represented increased by 17. The evolution of the 1000, 10,000-, and 15,000-compound libraries, on the other hand, saw a decrease by 15, 83, and 189 Murcko scaffolds respectively. This was while the number of unique predicted targets increased by 59 (to 423) for the 1000-compound library, and by 7 (to 991) for the 10,000-compound library, indicating that compounds with more promiscuous scaffolds have been selected through the course of evolution. The 15,000-compound library did see a reduction in the number of unique targets over the course of the evolution (by 81 targets, to 1281).

There is no change in the number of molecular clusters represented at the start and the end of the evolution for all the compound libraries. This is because the PCC forms over 24 thousand clusters, therefore the limiting factor in the number of clusters represented is the size of the library as each compound within a library is from a different cluster. Notably, every compound within a library is always from a different cluster, speaking to the diversity of the libraries.

Importantly, compounds selected during the evolution are in fact predicted to be bioactive towards newer targets more often. There are 59 more targets and 102 more Pfam families represented in the 1000-compound library at the end of 300 generations. The 5000 and 10,000-compound libraries also saw, as a result of evolution, a gain by 7 and 40 predicted targets, respectively, and 102 and 79 more Pfam families, respectively. For the 1000 and 10,000-compound libraries, the evolution resulted in a +5% (from 0.72 to 0.76) and +6% (from 0.71 to 0.75) respective change in the median novelty score of the targets predicted for the most fit individuals at the start and the end of the evolution. For the 5000-compound library, the median novelty score of the predicted targets was 0.75 for the fittest individual at both the beginning and the end of the evolution. An anomaly to this trend is observed for the 15,000-compound library where there is a reduction in the number of unique targets (by 81 targets to 1281) and consequently a very slight reduction in the median novelty score (from 0.75 to 0.74). There is, however, an improvement in the fitness of the 15,000-compound library, and this gain is acquired from the increase in predicted bioactivities (by 769 bioactivities to 25,139).

The optimization led to an increase in predicted bioactivities for all the compound libraries. That is, between the start and end of the evolution, the 1000-compound library has 395 (+24%) more predicted bioactivities, the 5000-compound library has 591 (+7%) more bioactivities, the 10,000-compound library has 805 (+5%) more bioactivities, and the 15,000-compound library has 769 (+3%) more activities. All these changes, coupled with the increase in the fitness score, show that the resulting compound libraries have got more predicted activity on novel targets.

**Figure 7:**
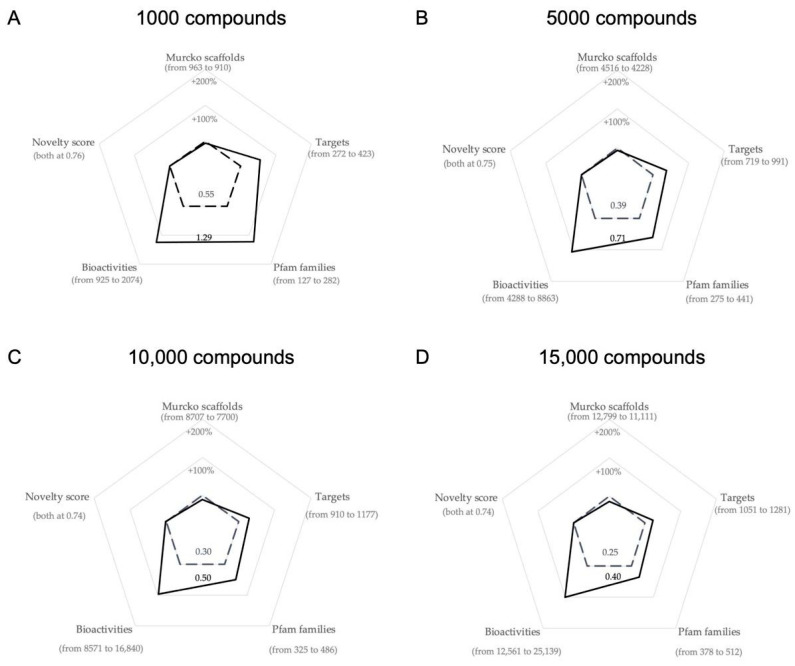
Radar charts visualizing the changes in the properties of the fittest library (solid black lines) of (**A**) 1000-compound library, (**B**) 5000-compound library, (**C**) 10,000-compound library, and (**D**) 15,000-compound library compared with the baseline populations (dashed black lines in each of the diagrams). The fitness values of the individual libraries are noted adjacent to the lines indicating the properties of the respective library.

Comparing these optimized compound libraries (Table 2) with the baseline compound libraries (Table 1), we see that the optimization shows remarkable improvements of the fitness across all libraries: +134% (0.55 vs. 1.29) for the 1000-compound library (Figure 7A), +82% (0.39 vs. 0.71) for the 5000-compound library (Figure 7B), +67% (0.30 vs. 0.50) for the 10,000-compound library (Figure 7C), and +60% (0.25 vs. 0.40) for the 15,000-compound library (Figure 7D). These improvements are driven by a steep increase in the number of predicted targets of the optimized compound libraries. When compared to the baseline compound libraries, between an additional 151 targets (+55% for the 1000-compound library) to 230 targets (+20% for the 15,000-compound library) are observed. Similarly, the number of bioactivities is also higher in the optimized libraries, between an additional 1149 bioactivities (+124% for the 1000-compound library) and 12,578 bioactivities (+100% for the 15,000-compound library), than the baseline libraries.

#### 2.2.3. Further Optimization of the Smaller-Sized Compound Libraries

As the smaller-sized compound sets were observed to benefit most from optimization by the genetic algorithm, we explored the possibility to further improve the sets of 1000 and 5000 compounds by re-running the genetic algorithm, this time with larger population sizes.

We first focus on the 1000-compound library. For this set, with a population size of 100 (Table 2), the optimization driven by the genetic algorithm yielded an increase in fitness by 34%. The larger population size of 500 (Table 3) led, over the course of its evolution, to an improvement in fitness by 46%, and with a population size of 1000, the improvement was 58% (Table 3).

Looking deeper at the 1000-compound libraries generated with population sizes of 100 (10% of the library size; Table 2) and 500 (Table 3), we see that the increase in final fitness values (1.29 vs. 1.42) correlates with an increase in the number of covered Murcko scaffolds (910 vs. 918), the number of targets (423 vs. 508), the number of covered Pfam families (282 vs. 303), and the number of predicted bioactivities (2074 vs. 2198). The increase in fitness is because the fitness function is designed to increase the score with repetitions in the predicted targets as the compound set is more likely to interact with a target when the target is predicted multiple times within the set.

The 1000-compound library generated with a population size of 1000 produced a 1000-compound library with the highest fitness score (1.56). The resulting 1000-compound library also covers more (930) scaffolds compared to 910 and 918 for the 1000-compound libraries generated with population sizes of 100 and 500, respectively. Clearly, during the course of this evolution, there is a slight increase in the number of scaffolds (from 928 to 930). This change occurred alongside an increase in the number of targets (from 433 to 710 targets from the start and end of the evolution) and Pfam families (from 198 to 323 Pfam families from the start and end of the evolution). As a result, despite only a slight increase in the number of represented scaffolds, the selected library is more populated with compounds that are predicted to interact with newer targets. This is also observed by the increase in the number of predicted bioactivities (from 1735 to 2743) and targets with higher novelty scores.

Considering the 5000-compound library, increasing the population size of the genetic algorithm from 500 individuals per generation (10% of the library size–Table 2) to 1000 and 5000 (Table 3) resulted in increased fitness of the best-scoring individuals (fitness of 0.71, 0.73 and 0.84, respectively). The improvement of the fittest individuals from the start and to the end of the evolution was also greater as the population size increased: a 20% improvement with a population size of 500, a 23% improvement when the population size was increased to 1000, and a 41% improvement for a population size of 5000. The fittest 5000-compound library generated from the population of 1000 individuals has an inverse relationship compared to the fittest library generated with the smaller population (500 individuals) on different fronts: a smaller number of scaffolds (4228 vs. 4193), fewer targets covered (991 vs. 917), and fewer Pfam families covered (441 vs. 412). The number of bioactivities, however, is higher (8863 vs. 8991), as is the median novelty score (0.75 vs. 0.76). This resulted in the improvement of the fitness score (0.71 vs. 0.73). Increasing the population size to 5000 resulted in a further increase in fitness for the 5000-compound library. Comparing the 5000-compound libraries generated with the populations of 1000 and 5000 individuals (with a fitness of 0.73 and 0.84, respectively), a different set of changes in the properties is observed with the increase in fitness. We still see that fewer and more promiscuous scaffolds are selected (4193 vs. 4175 Murcko scaffolds). However, this is coupled with more unique targets predicted (1062 vs. 917), more Pfam families covered (4210 vs. 470), and with more predicted bioactivities (8991vs. 10391) for the compound libraries. The different modulations of the properties to achieve higher fitness is a result of the multiple parameters which must be optimized.

**Figure 8:**
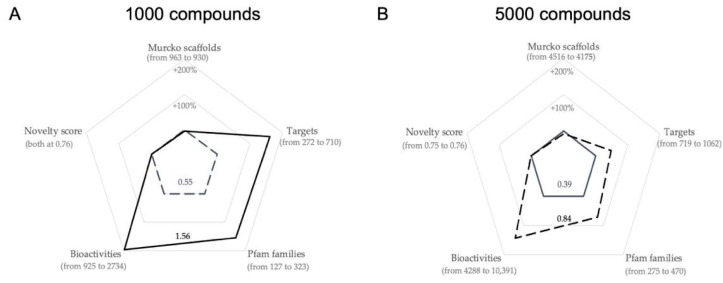
Radar charts comparing the change of properties between the baseline compound libraries and the further optimized compound libraries. The baseline compound libraries are depicted with dashed black lines for both the 1000-compound library generated with a population of 1000 ((**A**), black continuous line) and the 5000-compound library generated with a population size of 5000 ((**B**), black continuous line).

The 1000-compound library, which was optimized further with a population size of 1000, shows an improvement in fitness of 184% (0.55 vs. 1.56) over the baseline 1000-compound library (Figure 8A). This library also has an additional 438 (+161%) predicted targets, 194 (+154%) Pfam families and 1818 (+193%) predicted bioactivities compared to the baseline compound library. Likewise, the 5000-compound library, which was optimized further with a population size of 5000, has an improvement in fitness of 115% (0.39 vs. 0.84) over the baseline 5000-compound library (Figure 8B) and an additional 343 (+48%) predicted targets, 195 (+71%) Pfam families and 6103 (+142%) predicted bioactivities compared to the baseline compound library. These improvements show a clear benefit in optimizing compound libraries using this approach.

## 3. Materials and Methods

### 3.1. Data Sets

#### 3.1.1. ZINC20 Database

Via the ZINC20 web service [18,19], the 7,692,013 compounds annotated as “in-stock” [20] AND annotated as “anodyne” [20] AND assigned a charge state of −1, 0 or +1 AND assigned a calculated logP value between 0 and 4 were retrieved as SMILES strings from the ZINC20 database web service (Figure 1A) to be used as the source of compounds from which the optimized compound libraries would be generated.

#### 3.1.2. ChEMBL27 Database

From the ChEMBL27 database [22,23], the 2,156,988 bioactivity data records (i.e., compound-target pairs; Figure 1E) matching the following selection criteria (which are closely related to those used in previous works [21,27]) were retrieved:

1. Assay covers a single protein or a protein complex (ChEMBL confidence_score is 6, 7, 8, or 9)
2. data_validity_comment is null OR “manually validated”
3. potential_duplicate is “0”
4. standard_type is “Kd”, “Potency”, “AC50”, “IC50”, “Ki”, or “EC50”
5. activity_comment is not “Inconclusive”, “inconclusive”, or “unspecified”
6. NOT (standard_relation is null AND activity_comment is not “Active” or “active”)

Among the bioactivity records retrieved from the ChEMBL27 database, 4399 records had standard_units of “ug.mL^−1^” as opposed to “nM” and therefore the standard_value for these records was converted to nM using the canonical_smiles and RDKit’s Descriptors.ExactMolWt function.

### 3.2. Chemical Structure Processing and Data Consolidation

The chemical structures from both the ZINC20 and ChEMBL27 data (Figure 1B) were standardized using the ChEMBL Structure Pipeline [28] to remove salt components (note that the compounds from the ZINC20 database do not include salt components) and solvent components and to neutralize any charges. Only compounds with molecular weight between 250 and 900 Da and composed of C, H, O, N, P, S, F, Cl, Br, and I atoms were retained. The SMILES string of the canonical tautomer of the compounds, as obtained from RDKit’s TautomerEnumerator.canonicalize method, was recorded and used for further processing. In the case of the ZINC20 data, non-stereospecific SMILES were obtained and used to identify unique compounds based on their constitution (as information on the stereochemistry of the purchasable compounds is often incomplete or inaccurate).

Duplicate compounds in the processed ZINC20 data, resulting from the standardization process, were merged, resulting in 4,175,683 unique compounds. These ZINC20 compounds were further filtered for desirable molecular properties (see Molecular property filters for the ZINC20 data set).

In the case of ChEMBL27 data, when the standardization of the compounds resulted in duplicate compound-target pairs, the bioactivity records were merged and the median activity value of the merged records was set as the activity value for the compound-target pair. The 1,116,495 bioactivity records with activity values of less than or equal to 10,000 nM were labeled as “active” and were used as the reference data covering 661,839 compounds and 5170 targets for the similarity-based target prediction.

### 3.3. Filtering of the ZINC20 Subset by Molecular Properties

Following chemical structure processing, a series of molecular property filters (Figure 1C) were then used to remove any compounds with physicochemical properties that are unfavorable in the context of biochemical and cell research [11,17,29,30]. More specifically, any compounds matching any of the following criteria were removed:

1. Less than 18 or more than 30 heavy atoms (calculated using RDKit’s Lipinski.HeavyAtomCount method)
2. Less than one or more than four rings (calculated using RDKit’s CalcNumRings method)
3. Ring systems with more than three fused rings (calculated using RDKit’s GetRingInfo and AtomRings methods to get the ring systems and number of rings per system present a molecule)
4. More than eight rotatable bonds (rdMolDescriptors.CalcNumRotatableBonds)
5. More than three hydrogen bond donors (Lipinski.NumHDonors)
6. More than seven hydrogen bond acceptors (Lipinski.NumHAcceptors)
7. Charged carbon atoms (identified using RDKit atom properties)
8. Not at least one oxygen or nitrogen atom (identified using RDKit atom properties)
9. Substructures listed in the “remove” and “extreme caution” categories of the SMARTS patterns compiled by Chakravorty et al. [31]. These SMARTS patterns were compiled from a meta-analysis of existing structural filters to identify nuisance compounds and correctly identified 57% of noisy GSK compounds in the study’s validation [31]
10. Contain tosyl group (compounds which match the “S(=O)(=O)O” SMARTS pattern).

This filtering resulted in a final set of 2,572,351 ZINC20 compounds which were next subjected to target prediction.

### 3.4. Target Prediction

**Figure 9:**
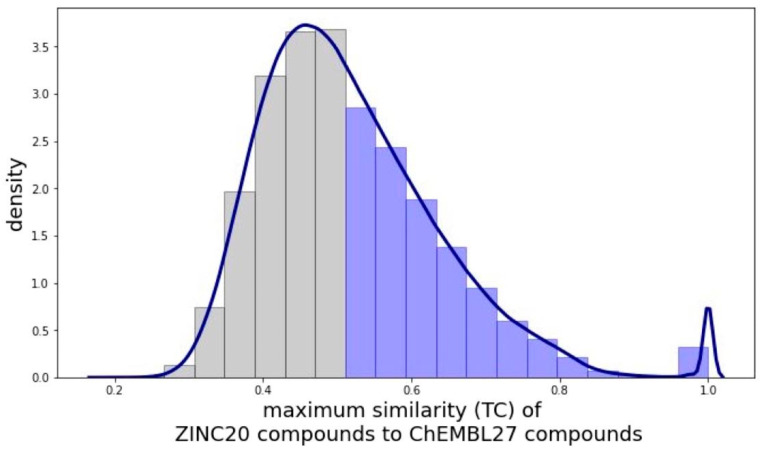
Distribution of the maximum similarities (quantified as Tanimoto coefficient based on Morgan fingerprints with radius 2 and length of 2048 bits) of the compounds derived from the ZINC20 data set to the compounds of the ChEMBL27 reference set for target prediction (derived from the ChEMBL27 database). The line is the kernel density estimate while the bars are the normalized histogram of the pairwise similarities. The distribution shows a large number of dissimilar pairs and a long tail as similarity increases. This observation is consistent with existing knowledge that two random compounds are more likely to be dissimilar than similar [33,34]. Of all the 2,572,351 compounds on which a similarity search was carried out, nearly half the compounds (1,257,596) had a maximum similarity of less than 0.5 to the ChEMBL27 reference set (grey bars). This means that for these compounds no likely targets could be identified by the computational approach. For the purpose of this study, these compounds were hence regarded as “dark chemical matter” [35], and since the aim of this study is to generate compound libraries with the best coverage of the target space, these compounds were discarded. The remaining 1,314,755 compounds (blue bars) were assigned the ChEMBL27 compounds’ targets as predicted targets. These 1,314,755 unique ZINC20 compounds had a coverage of 3362 predicted targets and were retained as the pool of candidate compounds (PCC) from which the final, optimized compound libraries will be generated with the genetic algorithm. The PCC had a median Tanimoto coefficient of 0.59 to the ChEMBL27 reference set and 32,032 compounds (2% of the PCC) had the same Morgan fingerprints as compounds in the ChEMBL27 reference set resulting in the peak at Tanimoto coefficient of 1.

The targets of compounds were predicted based on their 2D molecular similarity (Figure 1D) of the query compounds (i.e., all the 2,572,351 processed compounds from ZINC20) to any of the compounds in the ChEMBL27 reference set (i.e., all the 661,839 processed compounds from the processed ChEMBL27 database with their 1,116,495 bioactivity records covering 5170 targets). More specifically, this search was performed using Morgan2 fingerprints and the search.knearest_tanimoto_search_arena method implemented in chemfp [32]. Compound pairs with a Tanimoto coefficient of 0.5 or greater (Figure 9) were retained (as we found previously that for these compound pairs the probability of predictions to be correct is 60% or higher; see Figure 4 in Ref. [21]).

### 3.5. Additional Descriptions of the Compounds from the ZINC20 Database

In addition to the physicochemical properties that were used to filter the compounds from the ZINC20 database, Murcko scaffolds, QED score, logP, and compound clusters were used as additional descriptions of the compound sets. The number of Murcko scaffolds was calculated using the PandasTools.AddMurckoToFrame function of RDKit and counting the unique SMILES strings of the Murcko scaffolds. The QED scores were calculated using the rdkit.Chem.QED.default function of RDKit. The logP was calculated using RDkit’s Descriptors.MolLogP function. To cluster compounds, Dalke Scientific’s implementation [36] of the Taylor-Butina [37,38] clustering algorithm was utilized with Morgan 2 fingerprints and a Tanimoto threshold of 0.4.

### 3.6. Calculation of the Novelty of a Target

To map the diversity of targets, the targets were assigned to a Pfam family according to their protein sequence. Pfam is a large database of protein families that are represented by hidden Markov models which describe these families with the goal of increasing coverage with as few models as possible [24,25]. Sequences of the proteins in the ChEMBL27 reference set were retrieved from the ChEMBL27 database. The sequences were then searched against the library of Pfam hidden Markov models (Pfam-A.hmm; version 33.1) using the “Pfam_scan.pl” script (version 1.6) with default parameters and the “-clan_overlap” option to get a family classification (Figure 1G). All proteins for which no automatic assignment to a Pfam family could be obtained were assigned to a single “dummy” family. This is to account for the fact that, while these proteins were not assigned to a family by Pfam, and are thus different from the ones assigned to Pfam families, it is unclear how similar or different the unassigned proteins are to each other. Therefore, a conservative approach is to assign them to the same family and assume that they are similar. Bioactivity records, through their targets, were then labeled with a Pfam family.

To calculate the novelty score for the Pfam families, the dates when the bioactivity records were recorded needed to be retrieved. Wherever possible, these dates were retrieved from the ChEMBL27 database by linking an activity (using the activity_id) to its data source (the src.src_id and the docs.docs_id fields) and retrieving the year of publication (the docs.year field). For 343,389 of the bioactivity records, there was no date recorded in the ChEMBL27 database, and an attempt was made to find the relevant data when the primary data source (as recorded in the src_id field in ChEMBL database) was the PubChem Bioassay database [39] using the Assay ID (AID) which is also recorded in the ChEMBL database. Of the 1,360,528 bioactivity records (records before merging identical SMILES-target pairs) used for target prediction, 62,734 (i.e., less than 5%) did not have a date assigned as there was no date information recorded in the ChEMBL database for these records and these records had primary sources other than PubChem and dates could not be retrieved.

A novelty score (Equation (1)) for each Pfam family was then calculated. In the case of 1 of 1214 of the Pfam families where a score cannot be calculated (i.e., because the denominator could not be calculated due to a lack of dates for the bioactivity records), the average novelty score (0.71) of the scored families was assigned as the novelty score. The compounds from the PCC, along with their predicted targets, the target’s Pfam families, and target novelty scores, were then passed onto a genetic algorithm (Figure 1H) for subset selection, which was used to select an optimal subset of compounds for the compound libraries (Figure 1I).

### 3.7. Genetic Algorithm for Library Generation from the Pool of Candidate Compounds

A genetic algorithm was implemented to optimize the selection of compounds for the compound libraries from the PCC. In this implementation, an individual is defined as a set of N (where N = 1000, 5000, 10,000, and 15,000) compounds. A population, composed of M individuals, then evolves over generations to produce an optimal population containing the optimal (most fit) individual which is the selected library.

#### 3.7.1. Calculation of the Fitness Function

The fitness of an individual, that is a set of compounds, is determined by the diversity and the novelty of the targets with which the compounds are predicted to interact. The fitness score (Equation (2)) of an individual was calculated to capture the properties we aimed to maximize in the set selection: we want to optimize for a set of compounds that are predicted to interact with a diverse set of targets. To capture this, the score includes a summation of the novelty of each individual Pfam family represented in the set.

**Figure 10:**
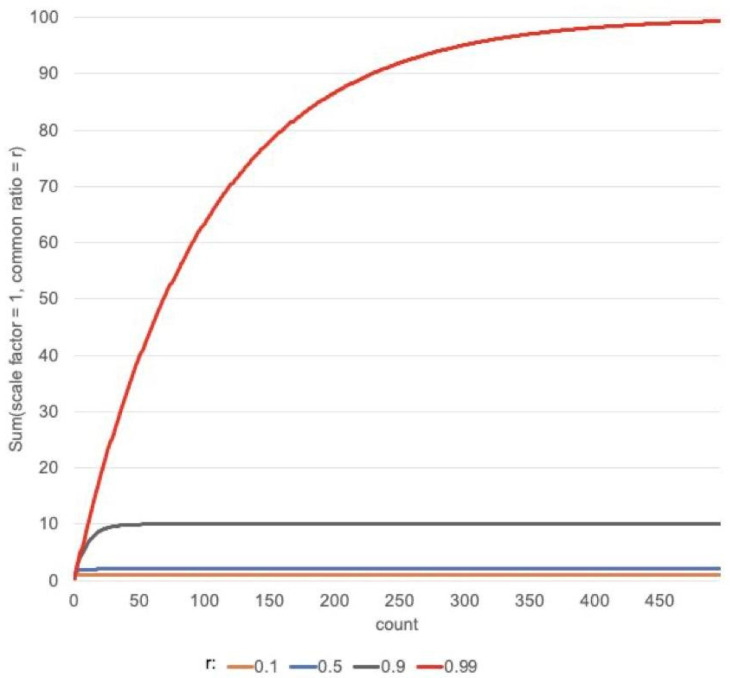
Sum of a geometric progression ($S=\frac{\mathrm{scale}\;\mathrm{factor}\left(1-r^{\mathrm{count}}\right)}{\left(1-r\right)}$) with a scale factor of 1 and varying values of the common ratio (r) versus the count. When used to calculate the fitness score, a sum of a geometric progression is calculated for each Pfam family (where the novelty score is set as the scale factor, and the number of times the Pfam family is predicted is set as the count) and summed to get the fitness score (Equation (2)).

When a Pfam family is represented multiple times in the predicted targets, the probability of a true interaction between the set of compounds and that family increases. Therefore, to capture the value of repeat predictions while still prioritizing diversity, the fitness function takes the form of a geometric progression (Equation (2)). This allows the score to increase with repeat family representation, however, the effect of the same family represented reduces with additional repeats. The scale factor of the geometric progression was set to the Pfam novelty score while the common ratio (r) was set to 0.99, as at this value of r a slow plateau in the sum is observed (Figure 10). The fitness score (Equation (2)) for an individual compound set is, therefore, the sum of the geometric sums of each of the Pfam families (where the count is the number of compounds predicted to interact with the family and the scale factor is the family’s novelty score), divided by the number of compounds in the individual.

#### 3.7.2. Library Optimization Procedure

**Figure 11:**
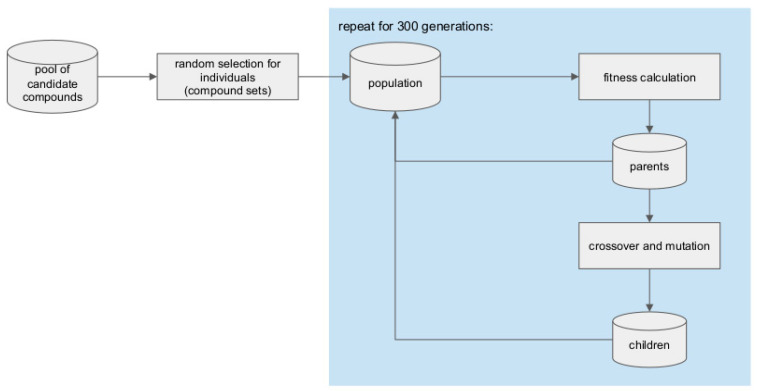
Schematic of the genetic algorithm which was implemented to select an optimal subset of compounds for the compound library.

The optimization of a compound library (Figure 11) begins by generating M (the population size) individuals, where each individual (i.e., compound set) is composed of N (the size of the compound library that is being generated) compounds randomly selected from the PCC. The fitness of each of the individuals in the population is calculated.

One third of the population size is composed of selected parents from the current generation. The fittest individuals of the current generation are selected as the parents. The remaining individuals (i.e., two-thirds of the population size) are children produced by mating the parents. Each pair of parents produces four children by passing on half of their compounds (as determined by a single point crossover in the middle of each parent) to each child. When a child inherits the same compound from both parents, one of the occurrences of the compound is mutated to a randomly selected compound ensuring that the new compound which is selected does not already appear in the child. Children are also mutated, to add variation, by randomly replacing 10% of the compounds with new compounds from the PCC. The parents and children are then pooled together to form the population of size M for the next generation whose fitness is evaluated. This process is repeated over 300 generations and the fittest individual at the end of the evolution is chosen as the optimal individual for a compound library of size N compounds. The parameters of N and M are detailed in Table 4.

## 4. Conclusions

In this study, we present a multi-step, computational approach for the design of small to medium-sized compound libraries that have a maximized likelihood of producing genuine hits in biological assays for an arbitrary target of interest. The approach takes multiple types of properties into account: drug-likeness, predicted bioactivities, biological space coverage, and target novelty. The hits identified by screening these compound libraries could serve as valuable tool compounds in biochemical and cell research, and some of them may also prove to be valid starting points for the development of drugs.

We have found that for all sizes of the compound libraries we generated (i.e., 1000, 5000, 10,000, and 15,000 compounds) the genetic algorithm improved the quality of the compound sets, with the individual libraries’ fitnesses improving up to 58%. The genetic algorithm was initially run with populations of 10% of the size of the library. As the smaller libraries (consisting of 1000 or 5000 compounds) benefitted the most from the optimization, further evolutions with larger population sizes were run, increasing the fitness of these libraries even more. In all cases, the objective fitness values, generated from the fitness function, increased through the course of evolution.

Multiple properties of the libraries were analyzed: number of Murcko scaffolds, the number of Taylor-Butina clusters, number of predicted targets, number of Pfam families of the predicted targets, number of predicted bioactivities, and the novelty scores of the predicted targets. These properties were modulated differently during the course of the optimizations to produce fitter libraries. In some cases, more diverse compound sets (as measured by a change in the number of Murcko scaffolds) were selected for the libraries which resulted in activity on a more diverse set of predicted targets. In other cases, compounds with more promiscuous scaffolds were selected which increased the number of targets they were predicted to be bioactive on. The modulation of these multiple objectives to produce better libraries highlights the appropriateness of our fitness function.

The benefits of utilizing target prediction and a genetic algorithm to optimize compound libraries are best seen when comparing fitness values of the optimized compound libraries with that of the baseline libraries (which do not account for predicted targets and have not been evolved). The largest improvement in fitness (+184%) was observed for the 1000-compound library generated with a population size of 1000, while the smallest improvement in fitness (+60%) was observed for the 15,000-compound library generated with a population size of 1500. The best of the optimized compound libraries prepared in this work are available for download as a dataset bundle (“BonMOLière”) from https://doi.org/10.5281/zenodo.5114733 (accessed on 19 July 2021).

## Figures and Tables

**Table 1:** Properties of the baseline compound libraries generated from compounds before predicted targets and the genetic algorithm are used to optimize the libraries.

Library Size	Fitness Score	Number of Murcko Scaffolds	Number of Targets	Number of Pfam Families	Number of Bioactivities	Median Novelty Score
1000	0.55	963	272	127	925	0.76
5000	0.39	4516	719	275	4288	0.75
10,000	0.30	8707	910	325	8571	0.74
15,000	0.25	12,779	1051	378	12,561	0.74

**Table 2:** Change in properties of the fittest individual (compound library) from the first generation to the fittest individual from after 300 generations generated with population sizes that were 10% of the library size ^1^.

Library Size	% Change in Fitness Score	Δ Number of Murcko Scaffolds	Δ Number of Compound Clusters Represented	Δ Number of Targets	Δ Number of Pfam Families	Δ Number of Bioactivities	% Change in the Median Novelty Score
1000	+34.48% (0.96→1.29)	−15 (925→910)	0 (1000 in both)	+59 (364→423)	+102 (180→282)	+395 (1679→2074)	+5.22% (0.72→0.76)
5000	+19.90% (0.59→0.71)	+17 (4211→4228)	0 (5000 in both)	+7 (984→991)	+102 (339→441)	+591 (8272→ 8863)	+0% (both at 0.75)
10,000	+16.89% (0.43→0.50)	−83 (7853→7770)	0 (10,000 in both)	+40 (1137→1177)	+79 (407→486)	+805 (16,035→16,840)	+6.11% (0.71→0.75)
15,000	+13.73% (0.35→0.40)	−189 (11,300→11,111)	0 (15,000 in both)	−81 (1362→1281)	−3 (515→512)	+769 (24,370→25,139)	−0.61% (0.75→0.74)

^1^ The “→” symbol indicates a change in the value of a characteristic between the fittest individual at the start and the end of the evolution.

**Table 3:** Change in properties of the fittest individual (compound library) from the first generation to the fittest individual from after 300 generations for the 1000-compound and 5000-compound libraries using different population sizes for the genetic algorithm ^1^.

Library Size	Population Size	% Change in Fitness Score	Δ Number of Murcko Scaffolds	Δ Number of Compound Clusters Represented	Δ Number of Targets	Δ Number of Pfam Families	Δ Number of Bioactivities	% Change in the Median Novelty Score
1000	500	+46.14% (0.97→1.42)	+6 (912→918)	0 (1000 in both)	+102 (406→508)	+101 (202→303)	+541 (1657→2198)	+2.26% (0.74→0.76)
1000	1000	+58.16%. (0.99→1.56)	+2 (928→930)	0 (1000 in both)	+267 (443→710)	+125 (198→323)	+1008 (1735→2743)	+0% (both at 0.76)
5000	1000	+23.12%. (0.59→0.73)	−59 (4252→4193)	0 (5000 in both)	+58 (859→917)	+15 (406→421)	+959 (8032→8991)	+1.33% (0.74→0.75)
5000	5000	+40.76% (0.60→0.84)	−17 (4192→4175)	0 (5000 in both)	+230 (832→1062)	+61 (409→ 470)	+2353 (8038→10,391)	+0.92% (0.75→0.76)

^1^ The “→” symbol indicates a change in the value of a characteristic between the fittest individual at the start and the end of the evolution.

**Table 4:** The population size parameters used in the genetic algorithms to optimize a library of size N (N = 1000, 5000, 10,000, and 15,000).

Library Size/Size of the Individual (N)	Population Size (M)
1000	100, 500, 1000
5000	500, 1000, 5000
10,000	1000
15,000	1500

## Data Availability

The best of the compound libraries presented in this work, including SMILES strings, ZINC IDs, and the ChEMBL IDs of the predicted targets), are available for download as a dataset bundle (“BonMOLière”) from https://doi.org/10.5281/zenodo.5114733 (accessed on 19 July 2021) [40].
